# Effects of geographic and economic heterogeneity on the burden of rotavirus diarrhea and the impact and cost-effectiveness of vaccination in Nigeria

**DOI:** 10.1371/journal.pone.0232941

**Published:** 2020-05-29

**Authors:** John D. Anderson, Clinton J. Pecenka, Karoun H. Bagamian, Richard D. Rheingans

**Affiliations:** 1 Goodnight Family Department of Sustainable Development, Appalachian State University, Boone, NC, United States of America; 2 Center for Vaccine Innovation and Access, PATH, Seattle, WA, United States of America; 3 Bagamian Scientific Consulting, Gainesville, FL, United States of America; 4 Department of Environmental and Global Health, University of Florida, Gainesville, FL, United States of America; Harvard University T H Chan School of Public Health, UNITED STATES

## Abstract

Child mortality from rotavirus gastroenteritis remains high in Nigeria, representing 14% of all rotavirus deaths worldwide. Here, we examine the potential impact and cost-effectiveness of national rotavirus vaccine introduction in geographic and economic subpopulations of Nigeria. We projected the health and economic outcomes of rotavirus vaccination in children over the first five years of life using a spreadsheet-based model. We modeled child populations using national survey data on rotavirus mortality risk factors and vaccination coverage to predict burden and impact across regional and wealth quintile subpopulations within Nigeria. Our base case considered introduction of a general rotavirus vaccine, modeled to encompass characteristics of existing vaccines, versus no vaccine. Base case costs were estimated from the government perspective, assuming Gavi subsidies, over the first five years. We also present estimates from the cost of vaccination from the perspective of Gavi. We explored uncertainty in model parameters through probabilistic uncertainty, one-way sensitivity, and scenario analyses. According to our estimates, rotavirus enteritis was responsible for 47,898 [95% Uncertainty Limits: 35,361; 63,703] child deaths per year, with approximately 80% of the national burden concentrated in the three northern regions of Nigeria. Rotavirus vaccination was estimated to prevent 6,454 [3,960; 9,721] deaths, 13% [9%; 18%] of the national annual RV burden. National ICERs for rotavirus vaccination from the Nigerian government and Gavi perspectives were US$47 [$18; $105] and $62 [$29; $130] per DALY averted, respectively. General rotavirus vaccination was projected to reduce rotavirus mortality by only 6% [4%; 9%] in the North West region compared to 35% [24%; 47%] in the South East region. Base case ICERs ranged from US$25 [10; 56] per DALY averted in North West to US$64 [18; 157] per DALY averted in South South. Gavi perspective ICERs ranged from US$33 [$15; $68] in North West to US$88 [35; 191] per DALY averted in South South. According to one-way sensitivity analyses, ICERs were most sensitive to vaccine efficacy, followed by estimated administrative costs and rotavirus mortality. Disparities in mortality reduction were largely driven by inequality in vaccination coverage across regions and between socioeconomic subpopulations. Due to high, persistent, and inequitable burden of rotavirus in Nigeria, routine vaccination with any of these rotavirus vaccines would be an high impact and cost-effective strategy in reducing child mortality.

## Introduction

Diarrhea is a leading cause of mortality among children under 5 years of age in Nigeria, causing approximately 103,000 deaths per year [1]. Over the past two decades, estimated diarrheal child mortality in Nigeria has declined from 803 deaths per 100,000 in 1997 to 303 deaths per 100,000 in 2017 [2]. While this 63% decline in rates over 20 years is a promising trend, the current rates remain nearly 50% higher than the rate of 205 diarrheal deaths per 100,000 children estimated for sub-Saharan Africa and are among the highest national estimates in the world [2].

While overall childhood diarrheal mortality has declined in Nigeria, burden of rotavirus gastroenteritis remains high and is the leading cause of childhood diarrheal mortality [1]. The Global Burden of Disease (GBD) study estimated rotavirus mortality rates had decreased from 285 per 100,000 children in 1990 to 173 per 100,000 in 2015, resulting in 55,474 rotavirus deaths in 2016 (GBD) [2]. The World Health Organization (WHO) estimated a decline from 255 deaths per 100,000 in 2000 to 101 deaths per 100,000 in 2013, resulting in 30,800 total deaths in 2013 [3].

The decline in child diarrheal mortality corresponds with improvements in important risk factors. The estimated fraction of children receiving treatment at a health facility increased from 22% to 41% from 2003 to 2008 Nigeria Demographic and Health Survey (NDHS) [4], before declining to 29% in the 2013 survey. Self-reported rates of use of oral rehydration solution (ORS) or recommended home fluids (RHF) increased from 22% in 2003 NDHS to 38% in 2013 NDHS [5,6]. However, the fraction of underweight children increased slightly (24% to 29%) and increases in children taken for treatment for diarrhea were modest between surveys.

Globally, more than 96 countries have introduced rotavirus vaccine, including many low- and middle-income countries [7]. In Africa, these introductions have resulted in documented reductions in hospitalizations and child mortality [8–10]. However, even though Nigeria accounts for the second highest number of rotavirus deaths by country—14% of all rotavirus deaths worldwide [3]—it has not yet introduced rotavirus vaccine. Our study is designed to support decisions about future rotavirus vaccine introduction by projecting potential impact and cost-effectiveness of a general rotavirus vaccine as compared to no vaccine in geographic and economic subpopulations within Nigeria. Characteristics of the general vaccine approximate the cost and delivery information of existing rotavirus vaccines. In other studies, geographic and economic factors have been shown to influence the potential risk of rotavirus mortality, economic burden of disease, and benefits of vaccination [11–13]. Our results provide important insights into where vaccination would have the greatest impact, be most cost-effective, and how improvements in routine vaccination strategies may further increase the benefits of rotavirus vaccine introduction.

## Materials and methods

### Overview

We estimated the expected health and economic outcomes of rotavirus vaccination compared to no vaccination for one annual birth cohort of children in Nigeria during the first five years of life using a spreadsheet-based deterministic model developed in Microsoft Excel [14]. We modeled the potential introduction of a general vaccine with parameters encompassing the characteristics of existing vaccines; ROTAVAC™ (Bharat Biotech), ROTASIIL™ (Serum Institute of India), and ROTARIX™ (GlaxoSmithKline Biologicals) that have received WHO prequalification, and ROTAVAC 5D™ (Bharat Biotech) and ROTASIIL LQ™ (Serum Institute of India) which are anticipated for WHO prequalification in 2020 [7,15–18]. We assumed that the general vaccine would be a three-dose vaccine delivered alongside diphtheria-pertussis-tetanus (DPT) vaccinations which are part of Nigeria’s Expanded Program on Immunization (EPI). We modeled the impact and cost-effectiveness of two-dose vaccines in sensitivity and scenario analyses.

We modeled rotavirus vaccination within a series of geographic and socioeconomic subpopulations, common sources of heterogeneity in diarrheal disease burden [1,19]. We used regional population estimates and child and household survey data on diarrheal mortality risk factors, patterns of care seeking, and access to vaccination from the 2013 NDHS [6]. In our analysis, we used the six regional administrative units in NDHS that represented all 36 states and the Federal Capital Territory, Abuja, as our geographic units of measure. Within each NDHS region, we grouped children into five wealth quintiles based socioeconomic status, defined by an asset index [20] using household data from NDHS [6]. In total, we modeled 30 regional socioeconomic subpopulations of children in Nigeria.

### Burden of rotavirus mortality

We calculated overall estimates of annual rotavirus mortality rates without rotavirus vaccination for children under five years old. Two commonly used sources of disease burden estimates report varying rotavirus mortality estimates for Nigeria. Therefore, we estimated mortality as the midpoint of the Institute for Health Metrics and Evaluation (IHME) GBD Study [21,22] and WHO [3] mean rotavirus mortality rates (deaths per 10,000 children, Table 1). Rates were cumulated from birth to estimate burden over the first five years of life.

**Table 1:** Rotavirus cost-effectiveness model input parameter values, distribution range, and vaccines used in uncertainty and sensitivity analyses. All costs are presented in 2019 US$.

INPUT	VALUE [RANGE*]	DISTRIBUTION	REFERENCE
Rotavirus Mortality			
Baseline cumulative national rotavirus mortality rate among children over the first five years of life (deaths/1,000 live births)	6.55 [5.35–8.30]	Lognormal (annual mean rate = 1.3, SD = 1.98)	Mean [3,23]
Cumulative Age Distribution			[24]
<3 months	3.0%	NA	
<6 months	15.8%	NA	
<12 months	57.6%	NA	
<24 months	96.6%	NA	
<60 months	100%	NA	
Risk Factors for Mortality			[6]
Oral rehydration treatment	93% effective	Binary, NA	[25]
Undernourished	Relative risk	1–12.5, NA	[26]
RV Vaccine—Efficacy			
Full Course (2 & 3 doses): Years 1–2	46.1% [29.1%– 59.1%]	Beta (alpha = 19.3, beta = 22.4)	[27,28]
Single dose reduced efficacy 2 dose	50% [38%– 63%]	Beta (alpha = 51.7, beta = 51.7)	Assumption
Single dose reduced efficacy 3 dose	33% [25%– 41%]	Beta (alpha = 76.1, beta = 153.4)	Assumption
Reduced efficacy in years 3–5	20% [0–40%]	Beta (alpha = 7.5, beta = 27.2)	Assumption
Vaccination			
DPT dose coverage (by subgroup)	Varied by region and quintile	NA	[6]
Vaccination timing (by subgroup)	Varied by region	NA	[6]
Medical Costs			
Mean medical cost per child (2019 US$)	$5.29	NA	[29]
Outpatient cost of illness	$6.80 [$5.02-$9.33]	Lognormal (SD = 1.1)	
Inpatient cost of illness	$57.61 [$42.57-$79.06]	Lognormal (SD = 20.8)	
Healthcare utilization by subgroup for relative medical costs			[6]
Pharmacy	$0.64	Binary, NA	[30]
Healer	$0.64	Binary, NA	[30]
Public (inpatient)	$71.00	Binary, NA	4 day stay [31,32]
Public (outpatient, rural)	$3.27	Binary, NA	[31,32]
Public (outpatient, urban)	$4.65	Binary, NA	[31,32]
Private (inpatient)	$91.55	Binary, NA	4 day stay [31,32]
Private (outpatient, rural)	$4.61	Binary, NA	[31,32]
Private (outpatient, urban)	$6.55	Binary, NA	[31,32]
Vaccine Costs			
Vaccine Price (per dose cost)			
General Vaccine	$1.08 [$0.85–$1.55]	Lognormal (SD = 0.14)	Assumption, [18]
ROTAVAC	$0.85 [$0.64–$1.40]	Lognormal (SD = 0.14)	[18]
ROTASIIL	$0.95 [$0.71–$1.55]	Lognormal (SD = 0.13)	[18]
ROTARIX	$2.29 [$1.72–$3.78]	Lognormal (SD = 0.39)	[18]
ROTAVAC 5D	$1.14 [$0.85–$1.58]	Lognormal (SD = 0.15)	[18]
ROTASIIL LQ	$1.55 [$0.95–$2.00]	Lognormal (SD = 0.25)	[18]
Administration (2019 US$)	$1.47 [$0.75–$2.00]	Lognormal (SD = 0.39)	[33]
Wastage			
General Vaccine	11% [4%-23%]	Beta (alpha = 9.3, beta = 68.3)	Assumption, [18]
ROTAVAC	23% [10%-41%]	Beta (alpha = 11.8, beta = 37.2)	[18]
ROTASIIL	10% [4%-14%]	Beta (alpha = 31.6, beta = 267.8)	[18]
ROTARIX	4% [3%-7%]	Beta (alpha = 22.4, beta = 514.2)	[18]
ROTAVAC 5D	10% [4%-14%]	Beta (alpha = 31.6, beta = 267.8)	[18]
ROTASIIL LQ	4% [3%-7%]	Beta (alpha = 22.4, beta = 514.2)	[18]

*Range of values explored in one-way sensitivity analysis. *‘NA’ indicates parameters that were not varied in sensitivity or uncertainty analyses*.

We modeled heterogeneity in rotavirus mortality risk by distributing the estimated national rate across economic and geographic subpopulations using an evidence-based risk index used in previous analyses [11,13,33,34]. This index assumes that a child’s susceptibility to diarrheal mortality is a function of their nutritional status based on published relative risk estimates for categories of weight-for-age z-scores [26] (*W*_*i*_) and relative risks estimates of receiving oral rehydration treatment (ORT) after a bout of diarrhea [25] (Eq 1). Each risk factor was estimated using 2013 NDHS data [6].

As oral rehydration data is only available for children with an episode of diarrhea in the previous 2 weeks, this limits the sample size for each subpopulation. To predict each child’s propensity for receiving ORT, we fit a logistic regression model with dependent variables of receiving either ORS or RHF after a diarrheal episode and independent, predictor variables that included demographic characteristics (e.g. household wealth quintile, maternal education, sex, region, and household residence, Tables 1 and 2) [11,33,34]. We estimated the probability of receiving ORT after experiencing diarrhea (*pO*_*i*_) for all children, using the PREDICT function in Stata (version 14) [35]. The individual risk factor for rehydration was calculated for each child as the product of their probability of receiving ORT and 7% (*β*_*o*_), based on 93% effectiveness of appropriate rehydration in preventing diarrheal mortality [25]. Individual mortality risk scores were aggregated for one-year-old children in each region (*r)* and wealth quintile (*q)* subpopulations.

$$I_{r,q}=\frac{\sum_{i}^{r,q}\beta_{o}∙{pO}_{i}∙W_{i}}{N_{r,q}}$$ Eq 1

**Table 2:** National and regional estimates of rotavirus burden, rotavirus vaccine effectiveness and economic impact for a cohort of children in Nigeria over the first five years of life.

	Burden (RV deaths / 1,000 births)	RV Deaths / year	RV Deaths Averted / year	Vaccine Benefits (RV deaths averted/1,000 births)	Vaccine % Reduction	Averted Costs of Illness (2019 US$ / 1,000 births)
NATIONAL	7.44 (5.49; 9.89)	47,898 (35,361; 63,703)	6,454 (3,960; 9,721)	1.00 (0.61; 1.51)	13 (9; 18)	1,066 (733; 1,428)
REGION						
North Central	5.98 (4.36; 7.98)	6,672 (4,865; 8,905)	1,102 (672; 1,650)	0.99 (0.60; 1.48)	17 (11; 22)	1,213 (825; 1,615)
North East	9.27 (6.84; 12.31)	9,654 (7,123; 12,815)	872 (539; 1,320)	0.84 (0.52; 1.27)	9 (6; 12)	349 (239; 468)
North West	11.54 (8.52; 15.29)	22,846 (16,864; 30,273)	1,460 (891; 2,181)	0.74 (0.45; 1.10)	6 (4; 9)	399 (271; 531)
South East	4.69 (3.44; 6.19)	2,478 (1,818; 3,269)	873 (535; 1,317)	1.65 (1.01; 2.49)	35 (24; 47)	1,637 (1,118; 2,180)
South South	3.43 (2.52; 4.54)	2,495 (1,838; 3,305)	714 (439; 1,076)	0.98 (0.60; 1.48)	29 (20; 38)	2,138 (1,463; 2,859)
South West	4.42 (3.25; 5.88)	4,634 (3,404; 6,156)	1,432 (880; 2,151)	1.37 (0.84; 2.05)	31 (21; 41)	2,313 (1,580; 3,069)

We estimated timing of projected deaths by combining overall rotavirus mortality estimates for each subpopulation and the estimated age distribution of diarrheal events from previous studies [24,36]. We estimated monthly rates for the first year of life and annual rates for the subsequent four years of life. To estimate rotavirus burden (*D*) for each subpopulation (*r*,*q*), we distributed national mortality rates (*M*_*n*_) across subpopulations by multiplying them by the normalized average risk scores for each subpopulation (*I*_*r*,*q*_, Eq 2.2). We then distributed the fraction of deaths (*A*) occurring within the first twelve months and then the first four years of life (*t*) by multiplying it by the normalized diarrheal mortality risk estimate for each subpopulation (*M*_*r*,*q*_, Eq 2.1).

$$D_{r,q,t}=A_{t}∙M_{r,q}$$ Eq 2.1

$${where,M}_{r,q}=\frac{M_{n}∙I_{r,q}}{\bar{I_{n}}}$$ Eq 2.2

To convert rotavirus mortality into disability-adjusted life-years (DALYs), we used standard DALY-estimation methods using age weighting and discounting [37]. We did not include DALYs associated with morbidity from acute cases in our estimates, because previous studies have shown that over 98% of DALYs associated with rotavirus diarrhea in low-income settings are associated with mortality [38,39].

### Vaccination coverage and effectiveness

We estimated rotavirus vaccination coverage for each region and wealth quintile subpopulation using DPT coverage data for one-year-olds surveyed during the 2013 NDHS [6]. To discern temporal trends in vaccination coverage disparities, we also calculated DPT coverage for one-year-old children from the two previous NDHS surveys (2003 [5] and 2008 [4]). Using regional NDHS data, we estimated the proportion of children receiving each dose by the end of monthly and annual time periods (*t*) and assumed uniform timing across quintiles due to data limitations. For each subpopulation, we defined coverage as the product of receiving a dose and the likelihood of receiving it within the recommended schedule.

We assumed that incomplete immunization of a child would have a reduced efficacy when compared to a child who received a full course for three-dose vaccines (33% less effective for each missed dose) and for a two-dose vaccine scenario (50% less effective per dose missed; Table 1). We conservatively assumed a 20% reduction in efficacy in years 3 to 5 for vaccinated children and tested this assumption in the sensitivity analysis [40]. Another assumption of this model is that efficacy against rotavirus mortality is equivalent to efficacy against severe illness, a common assumption in the absence of complete data on efficacy against mortality.

We assumed an efficacy of 46% for general and existing vaccines based on recent estimates of rotavirus vaccine efficacy in high-mortality settings two years after receiving the vaccine [27,28]. We estimated vaccine effectiveness for each subpopulation based on estimated coverage, expected timing, and efficacy of receiving each dose over time. We estimated vaccination benefits (*B*) for each region (*r*) and quintile (*q*) by combining estimate vaccination effectiveness (*E*) with expected DALY burden (*D*) over time (*t*) (Eq 3.1). Vaccination effectiveness was estimated as the product of the vaccine coverage (*V*) of each dose *d* and efficacy was defined as the incremental protection of each dose *d* during time period *t*.

$$B_{r,q,t}=D_{r,q,t}∙E_{r,q}∙H_{r,q}$$ Eq 3.1

$$\mathrm{where},\;E_{r,q}=e_{d,t}∙V_{d,r,q}$$ Eq 3.2

$$\mathrm{and}\;H_{r,q}=\frac{\bar{I_{r,q}∙v_{r,q}}}{I_{r,q}∙v_{r,q}}$$ Eq 3.3

We present health outcomes with rotavirus vaccination as the percentage of mortality averted (percent reduction) as well as the number and rates (per 1,000 children) of deaths averted by vaccination.

### Distribution and co-distribution of risk factors

While our calculations account for the correlation between individual risk and vaccine access at the region and quintile subgroup level, it also implicitly assumes that risk and access are not correlated within each subgroup. We tested this assumption by examining the correlation of community level DPT second dose (DPT2) coverage and mean risk index within each subgroup using Pearson’s correlation coefficients. To estimate DPT2 coverage, we aggregated individual coverage estimates to NDHS sampling clusters. We added a heterogeneity adjustment (*H*) as the mean product of estimated DPT2 coverage (*v*) and rotavirus risk index values (*I*) for each region (*r*) and quintile (*q*) divided by the product of coverage and risk for each region (*r*) and quintile (*q*) (Eq 3.3).

We further explored disparities in risk factors and access to vaccination in a series of logistic regression models that included determinants such as demographic, social, and geographic characteristics (e.g. sex of the child, socioeconomic status, maternal education, region, and residence) as predictors from the 2013 NDHS. Results of this analysis include summary means and standard errors for each determinant category and odds ratios with 95% confidence intervals (CI).

### Economic outcomes

Patterns of healthcare utilization and direct medical costs for diarrheal treatment vary geographically and by socioeconomic status. The average cost of outpatient and inpatient care per child for rotavirus diarrhea in Nigeria was extrapolated in a previously published study estimating diarrheal burden of other major enteric pathogens [29]. However, those estimates were originally based on research for rotavirus diarrheal costs. We distributed the estimated national direct medical costs per child across subpopulations using the mean relative cost-per-child estimates for diarrheal treatment after an episode of diarrhea in children 12 to 23 months of age from NDHS (Table 1). We combined proportions of children whose caretakers sought inpatient and outpatient care at public and private facilities from NDHS with WHO-CHOICE cost estimates [32]. We included costs of seeking informal care (traditional healer or pharmacy) by averaging published estimates from three African settings [30]. Since only a small fraction of children were reported to have an episode of diarrhea two weeks prior to participating in the NDHS, we imputed values for the rest of the surveyed children using a linear regression model predicting medical costs by region, wealth quintile, and household residence (urban versus rural setting) using Stata 14 statistical software [35].

We estimated costs averted by vaccination with each vaccine (*Ca*) for each region (*r*) and wealth quintile (*q*) as the product of estimated medical costs of treatment per child (*c*) and estimated vaccine effectiveness (*E*) in each subpopulation (Eq 4).

$${Ca}_{r,q}=E_{r,q}∙c_{r,q}$$ Eq 4

The base case for this analysis examines the introduction of a general rotavirus vaccine in Nigeria. The price of the vaccine was derived from Gavi, the Vaccine Alliance estimates [18], with a portion of doses being paid for by the Government of Nigeria and a portion by Gavi [41]. Our base case included the vaccine price from the perspective of the Government of Nigeria with Gavi support for planned introduction in 2020 (Table 1). We assumed Gavi price subsidies will require the Nigerian government to cover 44% of the price in 2020, increasing incrementally to 100% by 2029 [41]. Prices were projected by averaging the subsidized price over the first 10 years of rotavirus vaccination. We also calculated estimates from the Gavi procurement perspective, reflecting true societal costs of rotavirus vaccination in Nigeria (Table 1).

Our main economic outcome measures were incremental cost-effectiveness ratios (*ICERs*), estimated for each region (*r*) and quintile (*q*) subpopulation, defined as the incremental net costs divided by projected benefits (*B*, Eq 5) of rotavirus vaccination as compared to the counterfactual where rotavirus vaccines are not introduced in Nigeria. The incremental net cost of the intervention for each region and quintile was the sum of the estimated cost of administration and the price of each dose (*P*) minus the medical costs averted (*Ca*) for each child vaccinated in every region (*r*) and quintile (*q*) subpopulation. We assumed a wastage rate of 11%, ranging from 4% to 23%, based on the average and range of wastage rates from Gavi Detailed Product Profiles [18]. The estimated administration cost was $1.47 per dose [42] (Table 1). We estimated future costs and DALY outcomes in 2019 inflation-adjusted US dollars using changes in consumer price indices and discounted them at 3% [43].

$${ICER}_{r,q}=\frac{P_{r,q}-{Ca}_{r,q}}{B_{r,q}}$$ Eq 5

### Scenario, sensitivity, and uncertainty analyses

We used scenario analyses to estimate the cost-effectiveness of existing vaccines and better understand how improvements in vaccination coverage impact projected benefits. We analyzed pricing scenarios for five existing vaccines (ROTAVAC, ROTASIIL, ROTARIX, ROTAVAC 5D, and ROTASIIL LQ) to provide additional detail in S1 Table on variation in vaccine price and wastage assumptions. We simulated the potential impact of increasing vaccination coverage on ICERs by reducing proportions of unvaccinated children by 10% increments in every region using SimVoi [44].

We conducted a series probabilistic uncertainty and one-way sensitivity analyses to assess uncertainty in input variables and how those influenced predicted outcomes in SimVoi [44]. We assessed the effect of simultaneous variability in input variables using probabilistic uncertainty analysis. Key input variables were characterized as distributions (Table 1) and randomly sampled during Monte Carlo simulations for 10,000 model iterations to measure uncertainty in impact and cost-effectiveness estimates. Results are expressed as 95% uncertainty intervals (UI) around point estimates. We used one-way sensitivity analysis to estimate the effect of variation in individual input variables on ICERs calculated for each vaccine based on the range of each variable (Table 1). We explored uncertainty between two- and three-dose vaccines in our sensitivity analyses. Finally, we modeled scenarios of vaccination introduction with each of the prequalified vaccines along with ROTAVAC and ROTASIIL LQ to describe uncertainty in vaccination costs and delivery and included these results in S1 Table.

## Results

### Health burden

According to our estimates, rotavirus gastroenteritis caused 47,898 [UL: 35,361; 63,703] deaths per year over the first five years of life for children in Nigeria (Table 2). Within Nigeria, approximately 80% of the national burden was concentrated in the regions of North Central (NC), North East (NE), and North West (NW). The NW region had the highest estimated rotavirus burden, with an estimated 22,846 [UL: 16,864; 30,273] deaths per year attributed to rotavirus infection at a rate of 11.54 [UL: 8.52; 15.29] deaths per 1,000 births (Table 2). The South East (SE) region had the lowest burden, where rotavirus was estimated to cause 2,478 [1,818; 3,269] deaths per year. The lowest rotavirus mortality rate was projected for children living in the South South (SS) region (3.43 [2.52; 4.54] deaths per 1,000 births).

**Fig 1:**
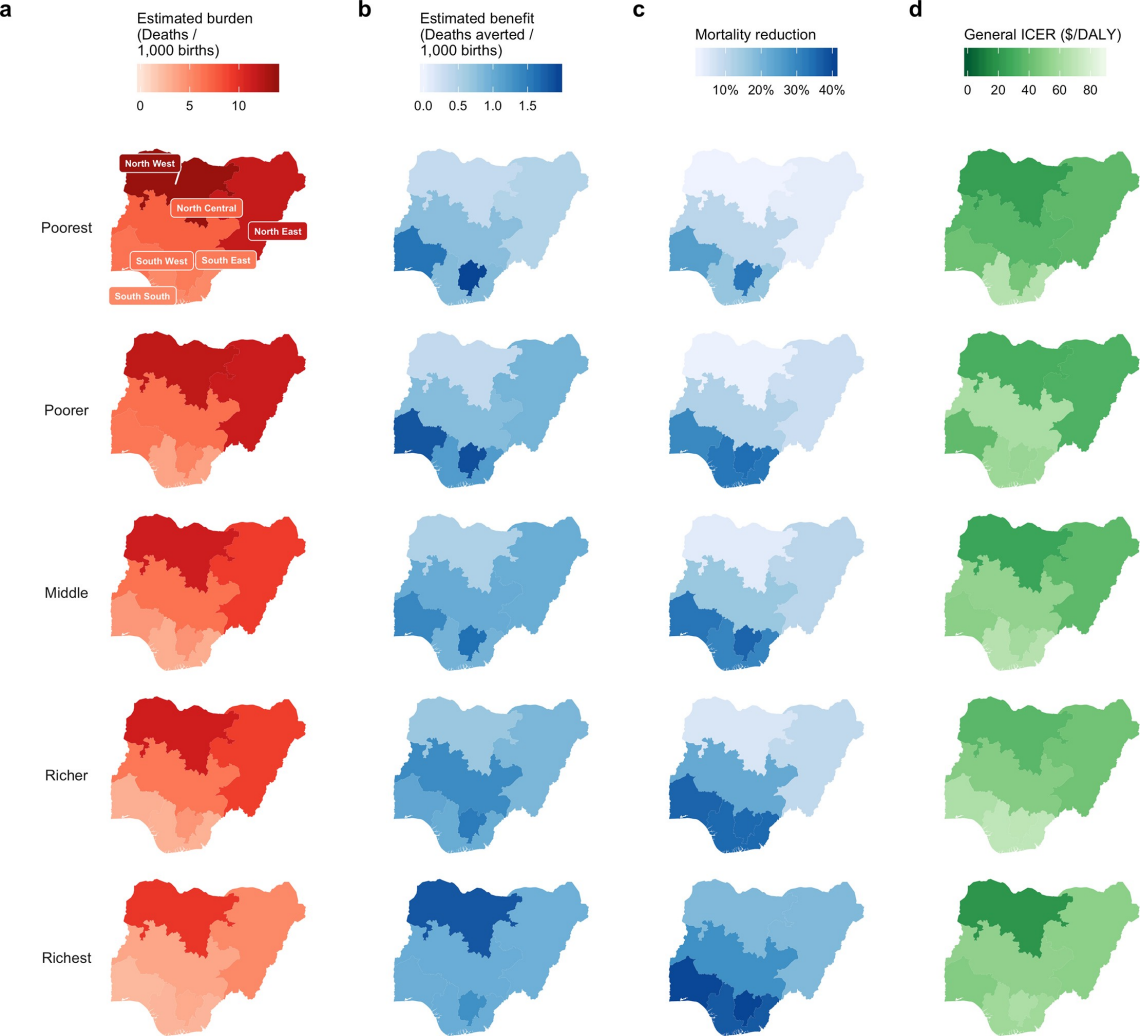
Estimated impact and cost-effectiveness of rotavirus vaccination by region and wealth quintile. Mortality burden (deaths, a) and benefit (deaths averted, b) are expressed as rates per 1000 births over the first five years of life. Benefit is also expressed as mortality reduction (c) which is the percentage of burden prevented by vaccination. Incremental cost-effectiveness ratios (ICERs) are displayed in green (d).

There was substantial heterogeneity in burden within each region (Fig 1A, S1 Table), with higher burden in the poorer quintiles in all regions and lower burden in wealthier quintiles. Populations of children from the poorer and poorest quintiles in NW (13.73 [10.13; 18.19] and 11.98 [8.84; 15.87] deaths per 1,000 births, respectively) had the highest rate of rotavirus mortality, followed by the poorest subpopulation in the NE (11.66 [8.61; 15.48] deaths per 1,000 births). The richest subpopulation of children from the SS region had the lowest rotavirus mortality rates (2.62 [1.93; 3.47] deaths per 1,000 births). The highest disparity in rotavirus mortality rates within a region was between the richest and poorest quintiles in NE (5.04 [3.72; 6.70] and 11.66 [8.61; 15.48] deaths per 1,000 births, respectively), followed by richest and poorest quintiles in the NW (9.53 [7.04; 12.64] and 13.73 [10.13; 18.19] deaths per 1,000 births, respectively). Rotavirus mortality risk was highest in the poorest subpopulations in all regions (Table 3).

**Table 3:** Mean susceptibility index scores for 1-year-olds in the 2013 NDHS. Estimates include summary means, standard errors (SE), and sample size (n) for each quintile and region.

		Wealth Quintile				
REGION		Poorest	Poorer	Middle	Richer	Richest
North Central	Mean	2.42	2.15	2.10	2.00	1.15
	SE	0.22	0.23	0.23	0.32	0.09
	n	121	160	143	153	199
North East	Mean	3.83	3.76	3.02	2.97	1.66
	SE	0.26	0.22	0.21	0.23	0.11
	n	176	193	220	194	175
North West	Mean	4.50	3.92	3.69	3.66	3.12
	SE	0.27	0.18	0.20	0.22	0.14
	n	288	306	276	273	254
South East	Mean	1.93	1.77	1.46	1.41	1.05
	SE	0.22	0.21	0.15	0.13	0.14
	n	101	96	112	101	85
South South	Mean	1.60	1.55	1.06	0.75	0.61
	SE	0.21	0.12	0.12	0.05	0.08
	n	138	134	146	127	94
South West	Mean	2.13	1.53	1.29	1.19	1.11
	SE	0.18	0.14	0.08	0.09	0.11
	n	146	139	125	117	120

According to our logistic regression models, disparities in child access to timely DPT vaccination and receiving ORT for child diarrhea across socioeconomic, demographic, and geographic subpopulations are important to understanding heterogeneity in diarrheal risk and burden (Table 4). When compared to children in the poorest quintile, children in the richest quintile had higher odds of receiving ORT after diarrhea and higher odds of being vaccinated for DPT1, DPT2, and DPT3 as well as receiving vaccination on time. There were also substantial geographic differences in both undernutrition and vaccination variables, with lower DPT1, 2, and 3 dose coverages in northern as compared to southern regions and higher odds of being of moderately-to-severely underweight children in NE and NW than in other regions.

**Table 4:** Logistic regression results with effects of predictive determinants on key risk and immunization dependent variables for 1-year-olds from the 2013 NDHS.

	DEPENDENT VARIABLES													
	DPT 2 (N = 5810)		DPT 2 (N = 5810)		DPT 3 (N = 5810)		DPT2 On Time (N = 1477)		Moderate to Severe Underweight (N = 5754)		Received ORT (N = 948)		Sought Treatment (N = 954)	
DETERMINANTS	Coverage	Logit (df = 816)	Coverage	Logit (df = 816)	Coverage	Logit (df = 816)	Proportion	Logit (df = 504)	Proportion	Logit (df = 816)	Proportion	Logit (df = 385)	Proportion	Logit (df = 385)
	% (SE)	OR (CI)	% (SE)	OR (CI)	% (SE)	OR (CI)	% (SE)	OR (CI)	% (SE)	OR (CI)	% (SE)	OR (CI)	% (SE)	OR (CI)
** Sex**														
Male	52.3 (1.4)	1.0	47.2 (1.4)	1.0	39.9 (1.3)	1.0	64.8 (2.3)	1.0	37.9 (1.1)	1.0	40.6 (2.8)	1.0	72.1 (2.7)	1.0
Female	48.8 (1.5)	0.9 (0.7, 1.0)	44.1 (1.5)	0.9 (0.8, 1.1)	37.4 (1.4)	0.9 (0.8, 1.1)	65.8 (2.3)	1.0 (0.7, 1.3)	35.1 (1.1)	0.9 (0.8, 1.0)*	42.7 (3.1)	1.1 (0.8, 1.6)	70.8 (2.8)	0.9 (0.6, 1.3)
** Wealth**														
Poorest	21.2 (1.7)	1.0	16.8 (1.6)	1.0	11.1 (1.3)	1.0	45.9 (6.0)	1.0	44.6 (1.8)	1.0	28.8 (3.8)	1.0	69.6 (3.7)	1.0
Poorer	29.6 (2.0)	1.4 (1.1, 1.8)*	24.9 (1.8)	1.4 (1.1, 1.9)*	19.0 (1.6)	1.6 (1.2, 2.2)*	53.4 (5.5)	1.2 (0.6, 2.2)	44.5 (1.8)	1.0 (0.8, 1.2)	39.6 (4.9)	1.5 (0.9, 2.6)	74.2 (4.0)	1.5 (0.9, 2.6)
Middle	50.7 (2.3)	2.0 (1.5, 2.6)*	44.6 (2.3)	2.0 (1.5, 2.7)*	36.2 (2.1)	2.3 (1.7, 3.1)*	54.2 (3.9)	1.0 (0.6, 1.9)	35.3 (1.9)	0.9 (0.7, 1.1)	41.8 (4.1)	1.5 (0.9, 2.5)	73.3 (3.6)	1.4 (0.8, 2.5)
Richer	74.2 (2.1)	3.2 (2.3, 4.5)*	67.7 (2.1)	2.9 (2.1, 4.1)*	59.1 (2.1)	3.4 (2.4, 4.8)*	62.6 (3.0)	1.3 (0.7, 2.4)	30.0 (1.9)	0.9 (0.7, 1.2)	54.3 (5.6)	2.2 (1.1, 4.1)*	71.0 (5.5)	1.4 (0.7, 2.9)
Richest	90.0 (1.2)	6.3 (4.1, 9.7)*	86.8 (1.3)	5.9 (3.9, 8.9)*	79.6 (1.6)	6.2 (4.2, 9.3)*	77.5 (2.4)	2.5 (1.3, 5.0)*	25.3 (1.7)	0.8 (0.6, 1.2)	63.8 (5.4)	2.8 (1.2, 6.3)*	66.2 (7.4)	1.5 (0.6, 3.8)
** Maternal education**														
No education	20.7 (1.3)	1.0	17.0 (1.2)	1.0	12.2 (1.1)	1.0	48.4 (4.2)	1.0	46.9 (1.2)	1.0	35.8 (2.7)	1.0	71.4 (2.8)	1.0
Primary	61.9 (1.9)	2.7 (2.1, 3.5)*	52.9 (2.1)	2.2 (1.7, 2.9)*	40.8 (2.0)	1.9 (1.5, 2.5)*	57.8 (3.4)	1.1 (0.7, 1.8)	33.6 (1.7)	0.9 (0.7, 1.1)	42.4 (4.2)	1.0 (0.6, 1.6)	73.1 (4.2)	1.2 (0.7, 1.9)
Secondary or higher	86.0 (1.0)	5.2 (3.9, 6.9)*	81.6 (1.2)	4.7 (3.5, 6.2)*	74.1 (1.3)	4.5 (3.3, 5.9)*	70.9 (1.9)	1.5 (1.0, 2.3)	23.7 (1.2)	0.6 (0.5, 0.8)*	52.4 (4.2)	1.2 (0.7, 2.2)	70.5 (4.4)	1.2 (0.6, 2.4)
** Region**														
North Central	61.9 (2.8)	1.0	55.0 (2.8)	1.0	44.2 (3.0)	1.0	59.6 (4.4)	1.0	26.3 (2.4)	1.0	38.6 (5.9)	1.0	81.9 (4.0)	1.0
North East	34.3 (2.6)	0.5 (0.4, 0.7)*	28.3 (2.4)	0.5 (0.4, 0.7)*	21.5 (2.1)	0.5 (0.4, 0.7)*	46.2 (4.2)	0.6 (0.4, 1.1)	40.0 (1.9)	1.6 (1.2, 2.1)*	38.0 (3.5)	1.1 (0.6, 2.1)	77.5 (2.6)	0.9 (0.5, 1.6)
North West	22.2 (1.8)	0.3 (0.2, 0.4)*	18.0 (1.7)	0.3 (0.2, 0.4)*	14.0 (1.5)	0.3 (0.2, 0.5)*	67.5 (4.8)	1.3 (0.7, 2.4)	51.1 (1.5)	2.5 (1.9, 3.3)*	40.3 (4.1)	1.2 (0.6, 2.2)	66.8 (3.9)	0.5 (0.3, 1.0)*
South East	89.5 (1.9)	2.4 (1.5, 3.8)*	87.6 (2.0)	2.6 (1.7, 4.0)*	81.8 (2.3)	2.8 (2.0, 4.0)*	74.4 (3.3)	1.4 (0.8, 2.3)	20.4 (2.1)	0.8 (0.6, 1.2)	43.0 (6.2)	0.8 (0.4, 1.7)	63.7 (7.7)	0.4 (0.1, 0.9)*
South South	85.3 (2.2)	1.6 (1.1, 2.4)*	80.3 (2.5)	1.6 (1.1, 2.2)*	71.0 (2.7)	1.5 (1.1, 2.1)*	66.5 (3.7)	1.0 (0.6, 1.6)	18.3 (1.8)	0.8 (0.5, 1.0)	57.2 (7.8)	1.7 (0.8, 4.0)	77.8 (7.7)	0.7 (0.2, 2.0)
South West	81.7 (2.4)	1.0 (0.6, 1.4)	76.4 (2.5)	0.9 (0.6, 1.3)	66.0 (2.7)	0.8 (0.6, 1.1)	67.4 (3.4)	0.7 (0.4, 1.1)	29.2 (2.0)	1.3 (1.0, 1.8)	56.2 (6.9)	1.0 (0.5, 2.1)	55.1 (9.9)	0.2 (0.1, 0.7)*
** Setting**														
Urban	73.9 (1.8)	1.0	70.0 (1.8)	1.0	62.7 (1.9)	1.0	73.6 (2.0)	1.0	30.9 (1.3)	1.0	54.5 (3.2)	1.0	68.2 (3.7)	1.0
Rural	37.6 (1.5)	0.9 (0.7, 1.2)	32.1 (1.4)	0.8 (0.6, 1.0)*	25.3 (1.3)	0.8 (0.6, 0.9)*	53.2 (2.6)	0.6 (0.4, 0.8)*	39.8 (1.1)	0.9 (0.8, 1.1)	35.5 (2.7)	0.7 (0.4, 1.0)	73.0 (2.4)	1.1 (0.7, 1.9)
**Constant**	-	0.4 (0.3, 0.7)*	-	0.4 (0.3, 0.6)*	-	0.2 (0.1, 0.3)*	-	1.2 (0.6, 2.7)	-	0.5 (0.4, 0.8)*	-	0.5 (0.2, 1.2)	-	2.9 (1.3, 6.8)*

Estimates include summary means and standard errors (SE) for each determinant category and odds ratios (OR) with 95% confidence intervals (CI) in parentheses and significant results (alpha less than or equal to 0.05) denoted by asterisks (*). Risk variables include being moderately-to-severely underweight, defined as less than -2 standard deviations below median weight-for-age of reference populations, receiving oral rehydration treatment (ORT), either oral rehydration solution or recommended home solution, for a bout of diarrhea that occurred within 2 weeks of the 2013 NDHS. Immunization variables include receiving one, two, or three doses of DPT and receiving the second dose of DPT on time.

Correlations between community estimates of diarrheal risk factors and immunization at the national and regional levels support the use of our heterogeneity adjustment parameter. Immunization coverage (both DPT1 and DPT2) are positively and moderately correlated with use of ORT positively and weakly correlated with weight-for-age z-scores at the national level (Table 5). Positive and moderate correlations between community DPT2 coverage with ORT are present in all regions as well, though weaker in the SE and SW where DPT 1 and 2 dose coverage is higher.

**Table 5:** Community level Pearson’s correlation coefficients of mortality risk factors and DPT coverage.

Risk factors	Correlates	National	North Central	North East	North West	South East	South West	South South
DPT 1	DPT 2	0.95*	0.91*	0.95*	0.95*	0.81*	0.82*	0.87*
	DPT2 Ontime	0.19*	0.11	-0.12	0.36*	0.17	0.00	0.19*
	WFA z-score	0.42*	0.31*	0.20*	-0.16*	0.09	0.12	0.09
	Probability ORT	0.59*	0.56*	0.46*	0.63*	0.22*	0.31*	0.53*
DPT 2	DPT2 Ontime	0.20*	0.09	-0.05	0.37*	0.19*	0.06	0.16
	WFA z-score	0.41*	0.30*	0.24*	-0.21*	0.09	0.01	0.11
	Probability ORT	0.59*	0.60*	0.49*	0.60*	0.24*	0.31*	0.52*
DPT 2 Ontime	WFA z-score	0.19*	0.15	0.10	-0.17	0.33*	0.09	0.19*
	Probability ORT	0.27*	0.34*	0.24*	0.35*	0.37*	0.19*	0.23*
WFA z-score	Probability ORT	0.34*	0.28*	0.29*	-0.16*	0.20*	0.28*	0.15
	N	889	0	134	182	116	140	155

Statistical significance is notated with an asterisk (*) when p-values were less than or equal to alpha = 0.05.

### Economic burden

**Fig 2:**
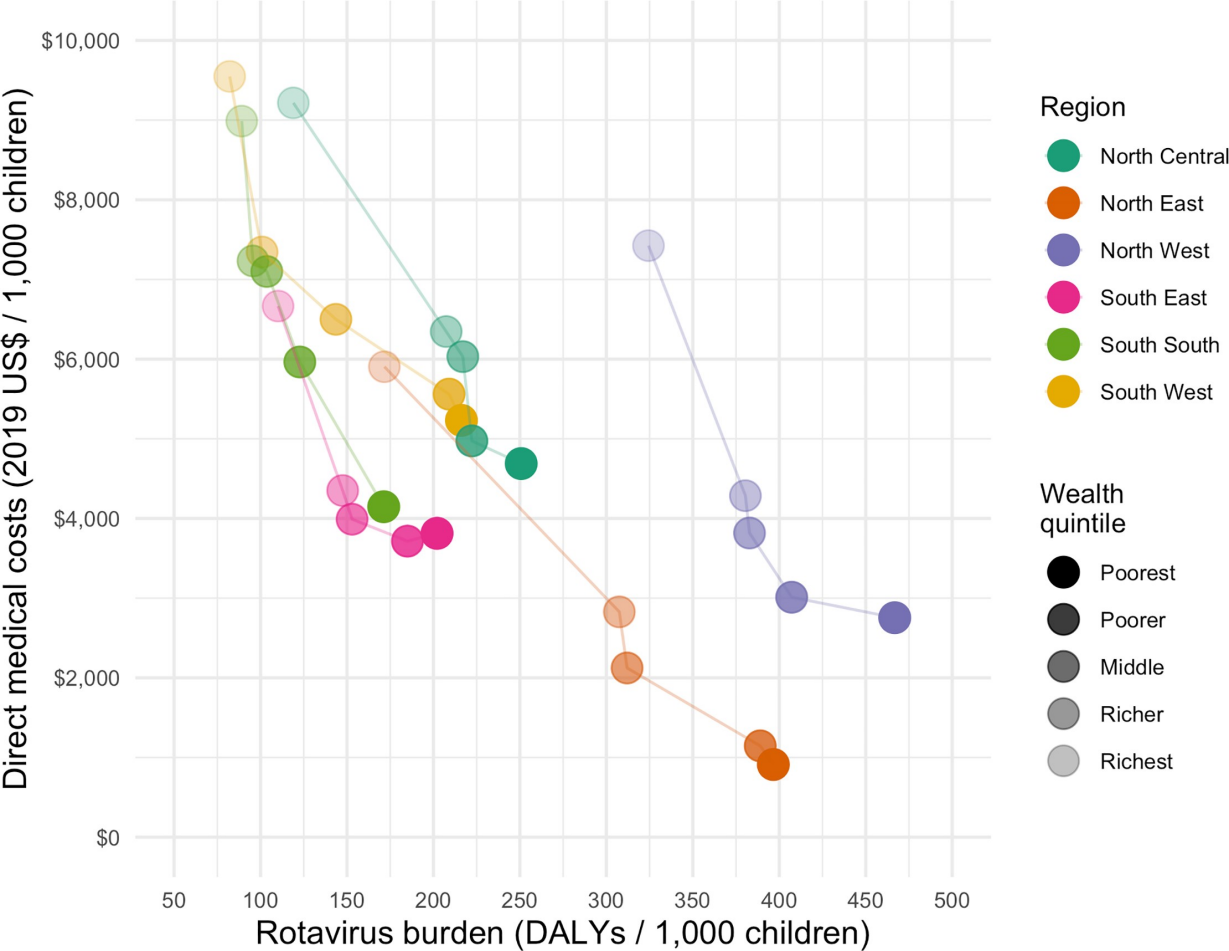
Estimated direct medical costs of rotavirus vaccination and rotavirus mortality burden across regional quintiles in Nigeria during the first five years of life.

There was an inverse relationship between health and economic burden in all regions: economic burden increased with wealth, while health burden decreased with wealth (Fig 2). While NE and NW had the highest estimated rotavirus health burden, NE and SE had the lowest estimated household economic burden. The burden of direct medical costs was higher in the richest quintiles, where rotavirus burden is lower. The largest disparities in rotavirus direct medical costs and disease burden were both in NE (Fig 2).

### Impact of vaccination

**Fig 3:**
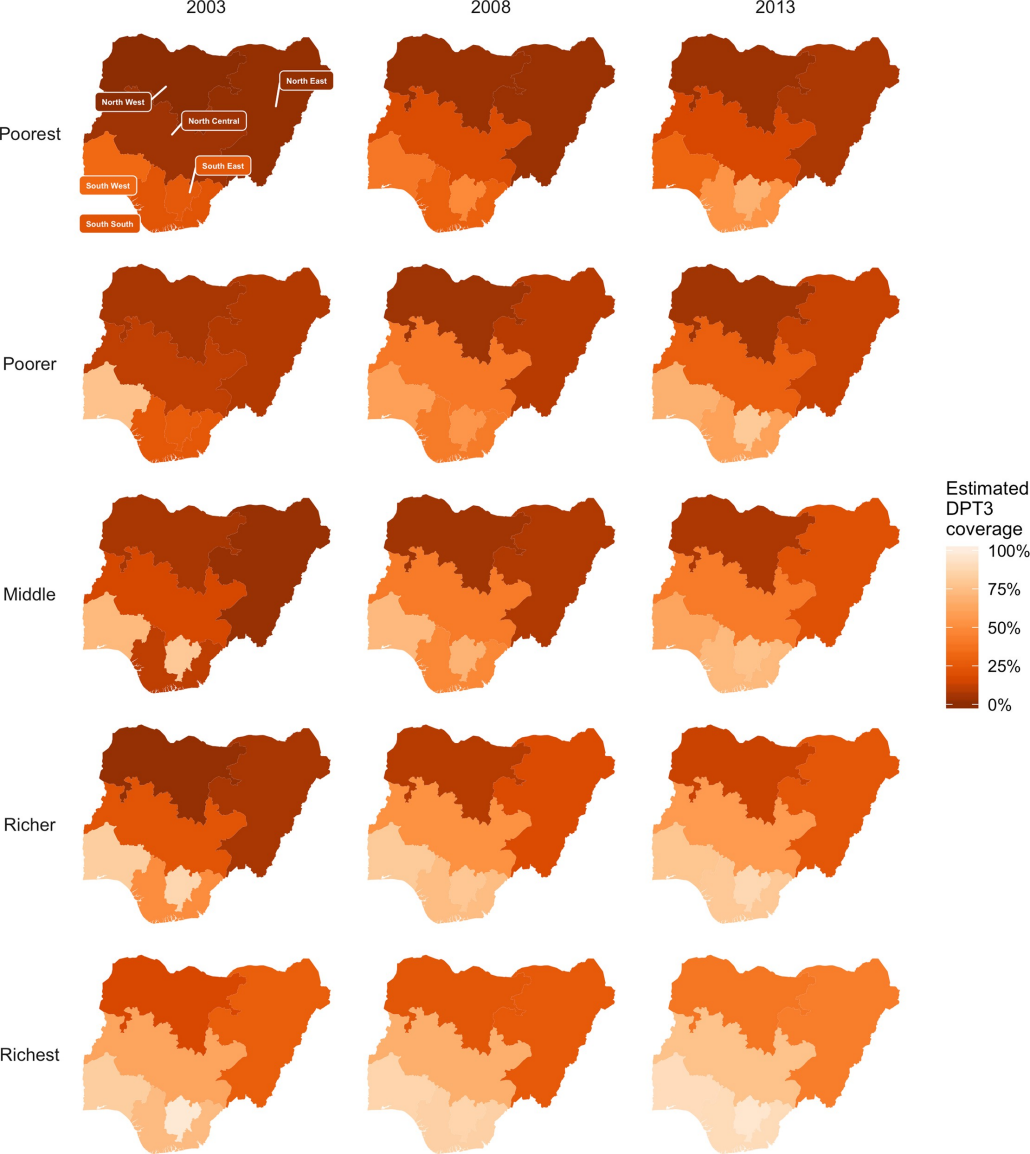
Estimated DPT 3 vaccination coverage, used as a proxy for estimating a 3-dose rotavirus vaccination schedule for each regional wealth quintile across the most recent three NDHS: 2003, 2008, and 2013. Darker orange indicates lower vaccination coverage while lighter orange is closer to full coverage.

Based on rotavirus coverage estimates from 2013 (Fig 3), rotavirus vaccination introduction would prevent 6,454 [3,960; 9,721] deaths, 13% (9%; 18%) of the total annual RV burden in Nigeria (Table 2). The greatest absolute benefits of rotavirus vaccination were among children living in the NW (1,460 [891; 2,181] RV deaths averted, Table 2). In the southern regions, estimated percent reduction of annual burden ranged from 29% [20%; 38%] in the South South (SS) region to 35% [24%; 47%] in SE, while the percent burden reduction in the Northern regions were much lower (ranging from 6% [4%; 9%] in NW to 17% [11%; 22%] in NC; Table 2).

According to the past three NDHS surveys, northern Nigeria has had less improvement in DPT3 coverage over the past decade than was reported in the southern regions (Fig 3). Projected rotavirus vaccination coverage (2013 DPT3 coverage) was lower in the poorest populations than in the richest populations. Proportions of unvaccinated children were highest in the NE and NW regions, where estimated burden was also the highest and concentrated in the poorest populations. Within regions, the highest benefit was found in the poorest and poorer quintiles in SS and SW and in the richest quintile in NW (Fig 1B). Mortality reductions were highest for children in the richest quintiles in the three southern regions (Fig 1C).

### Cost-effectiveness of vaccination

**Fig 4:**
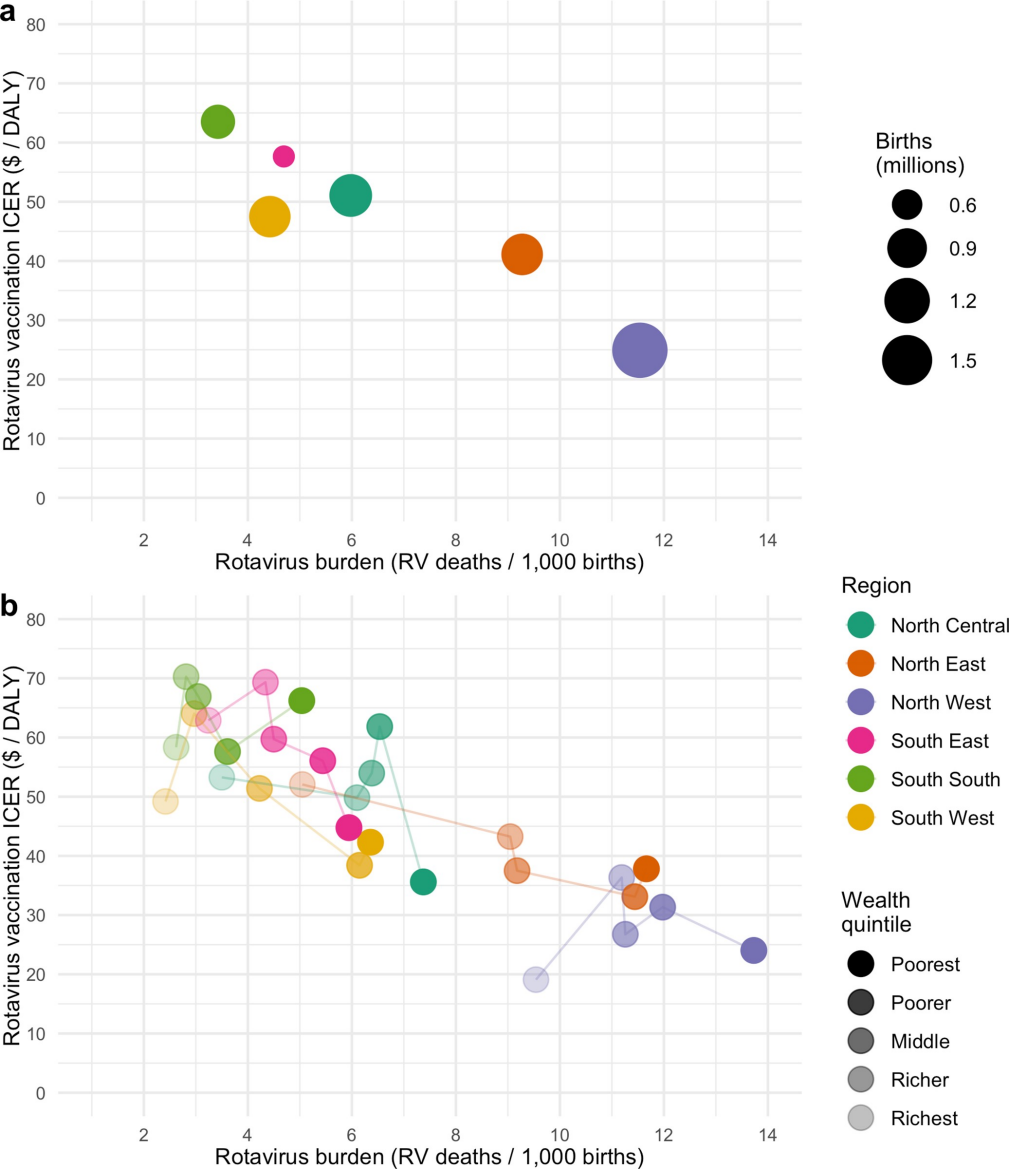
Estimated incremental cost-effectiveness ratios (ICERs) for rotavirus vaccination by rotavirus mortality burden for each region (a) and wealth quintile (b) subpopulation.

The national estimated ICER for rotavirus vaccination from the Nigeria government perspective was US$47 [$18; $105] per DALY averted (Table 6) as compared to the no rotavirus vaccine counterfactual. The estimated national ICERS from the Gavi perspective was US$62 [$29; $130] per DALY averted. Incremental cost-effectiveness ratios from the Nigeria government perspective were lowest in the NW (US$25 [$10; $56] per DALY averted) and NE (US$41 [$21; $82] per DALY averted) regions, with the highest ICER in the SS region (US$64 [$18; $157] per DALY averted, Table 6). All regional ICERs had an inverse relationship to burden, where lower ICERs (thus more favorable) were found in higher rotavirus mortality burden regions (Fig 4A and S6A–S6E Fig). ICERs varied within regions, with similar inverse relationships with rotavirus mortality burden in many regions. However, this pattern of inverse relationships in regional ICERs does not hold for the richest quintiles in SE and SW and for the poorer, middle and richer quintiles in NC, NW, and SS (Fig 4B, S6F–S6J Fig). For NC, NW, and SS regions, ICERS were higher for children from the poorer, middle, or richer quintiles than children from the richest quintile.

**Table 6:** Estimated national and regional vaccination costs and cost-effectiveness of rotavirus vaccine introduction in a cohort of children in Nigeria over the first five years of life. ‘Average Nigeria perspective’ incremental cost-effectiveness ratio (ICER) base case estimates assumed Gavi subsidies for the first 5 years of vaccine introduction. ‘Full price’ is the cost of the vaccine from the perspective of Gavi.

	Average Nigeria Perspective Vaccination Costs (2019 US$ / 1,000 births)	Gavi Perspective Vaccination Costs (2019 US$ / 1,000 births)	Average Nigeria Perspective ICER (2019 US$ / DALY averted)	Gavi Perspective ICER (2019 US$ / DALY averted)
NATIONAL	2,667 (1,924; 3,760)	3,185 (2,389; 4,291)	47 (18; 105)	62 (29; 130)
REGION				
North Central	2,927 (2,108; 4,120)	3,495 (2,626; 4,719)	48 (19; 116)	68 (31; 144)
North East	1,520 (1,098; 2,123)	1,815 (1,355; 2,439)	41 (21; 82)	51 (28; 98)
North West	1,024 (733; 1,441)	1,223 (910; 1,649)	25 (10; 56)	33 (15; 68)
South East	4,877 (3,519; 6,834)	5,824 (4,356; 7,886)	58 (26; 122)	74 (37; 149)
South South	4,253 (3,060; 5,982)	5,078 (3,798; 6,896)	64 (18; 157)	88 (35; 191)
South West	4,519 (3,240; 6,390)	5,397 (4,055; 7,305)	47 (12; 118)	66 (26; 148)

### Scenario and sensitivity analyses

**Fig 5:**
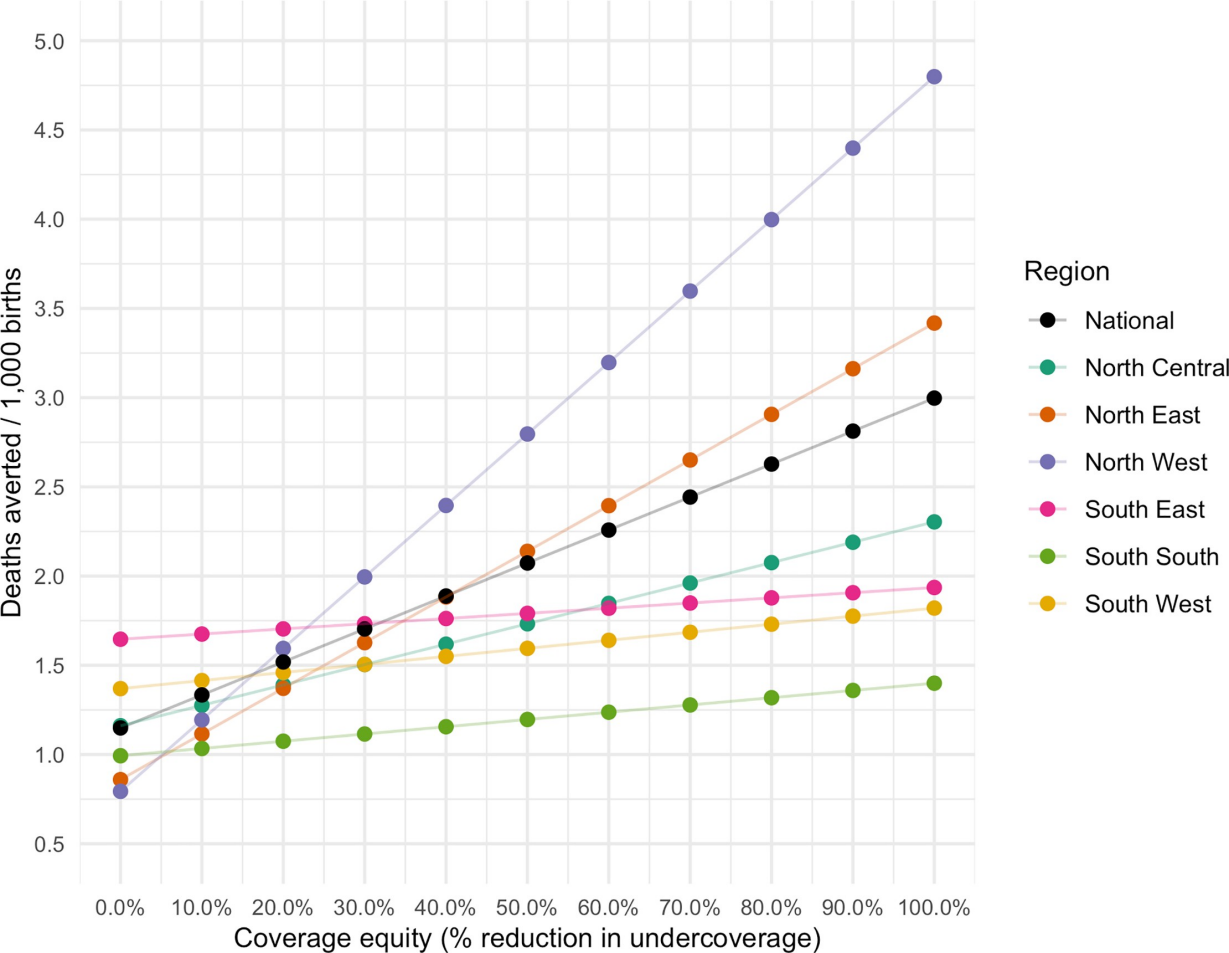
Results of a scenario where the benefits (deaths averted per 1,000 births) are estimated in response to incremental 10% reductions in the percentages of unvaccinated children (undercoverage) in each region.

Improvements in coverage had the largest effects in the NE and NW regions, where estimated rotavirus vaccine coverage was the lowest. A 50% improvement in coverage would result in deaths averted per 1000 rates that are 3.5 and 2.5 times higher in NW and NE, respectively (Fig 5). Nationally, full and equitable coverage would have the greatest effects in the most vulnerable regions with a 149% increase over baseline estimates, preventing 19,580 deaths annually (41% of annual estimated rotavirus deaths).

**Fig 6:**
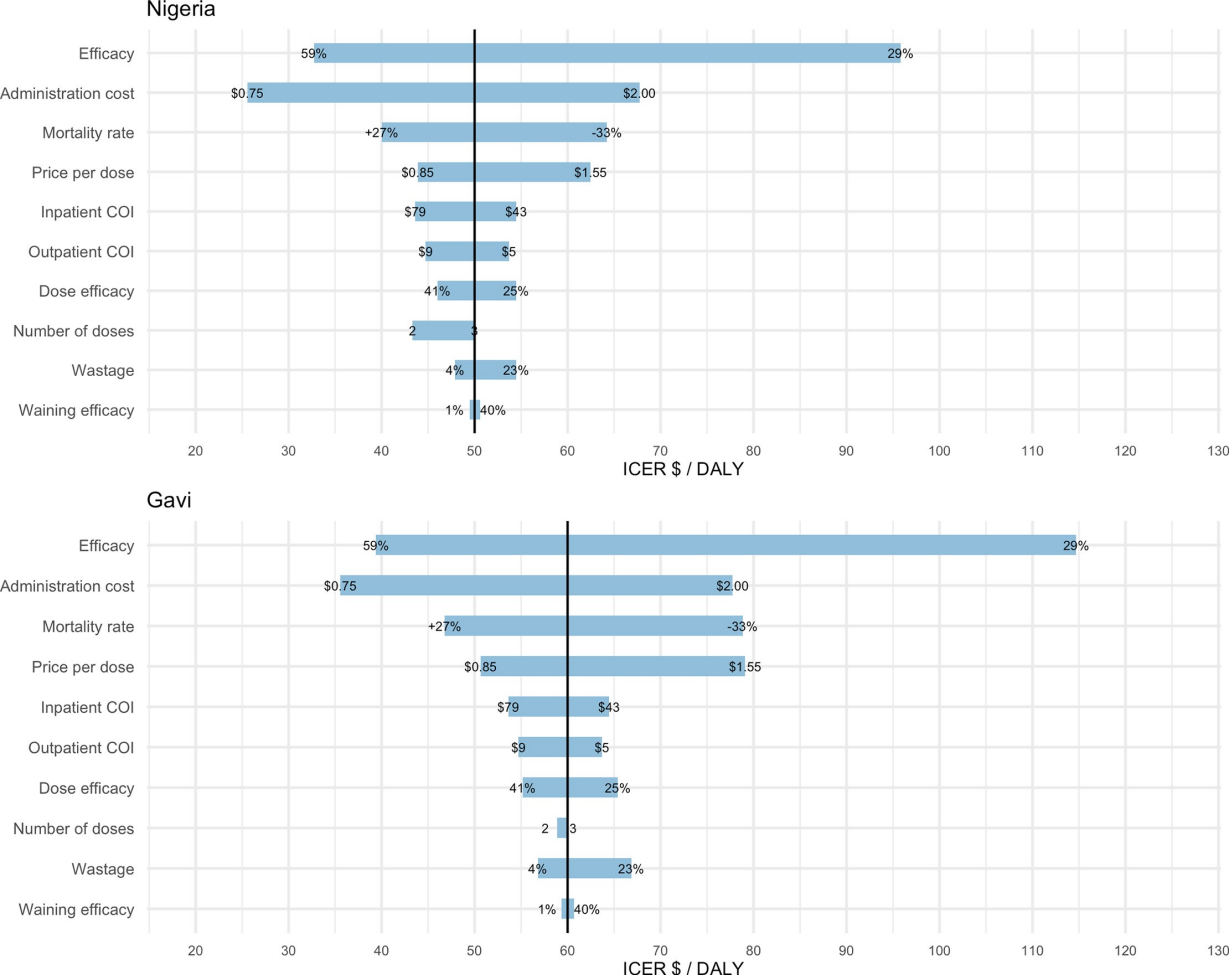
Tornado diagram showing the results sensitivity analysis for vaccination with a general rotavirus vaccine at prices from the Nigerian government perspective (Nigeria) and the Gavi perspective (Gavi). Each input variable and tested assumption is listed on the left axes with corresponding simulated value ranges displayed at the end of each bar.

Uncertainty in vaccine efficacy had the greatest effect on estimated Nigerian government and Gavi perspective ICERs in the one-way sensitivity analysis with swings of $63 and $75 per DALY (Fig 6). Following efficacy, uncertainty in administrative costs was the next most significant variable with swings of $42 per DALY in both Nigerian and Gavi perspective ICERs. The third most influential variable on uncertainty was rotavirus mortality rate estimates which accounted for swings of $24 and $32 per DALY for Nigerian government and Gavi perspective ICERs, respectively. Uncertainty around vaccine price was the fourth most influential, causing swings of $19 and $28 per DALY for Nigerian government and Gavi perspective ICERs, respectively.

## Discussion

Due to the high, persistent, and inequitable burden of rotavirus in Nigeria, routine vaccination within the price range of current and future WHO prequalified rotavirus vaccines would be a highly impactful and cost-effective strategy in all subpopulations and regions of Nigeria. We found that patterns of vaccination and rotavirus disease risk in Nigeria are strongly affected by geography, socioeconomic level, and household characteristics. Estimated vaccine coverage was lower, and risk of disease was higher for children from northern regions, rural areas, and poorer households. In addition, we found that low coverage and disease risk factors correlated at community levels.

Our projections showed that the highest potential impact of rotavirus vaccination would be in the most vulnerable subpopulations of children where rates of rotavirus mortality are the highest. However, the poorest subpopulations in northern regions were predicted to have lower rates of mortality reduction from vaccination than was predicted for the poorest in southern regions, despite northern subpopulations having higher mortality rates southern subpopulations. Disparities in mortality reduction were largely driven by much lower estimates of vaccination coverage in northern as compared to southern regions. Despite having the highest underlying burden, few children in these populations are predicted to be vaccinated, resulting in the lowest estimated mortality reduction across all subpopulations. Improving equity in coverage would greatly increase health gains, maximizing the value of rotavirus vaccination in Nigeria.

Reductions in proportions of unvaccinated children require interventions targeting barriers to routine vaccination, particularly in the most vulnerable populations throughout Nigeria, to maximize the potential of rotavirus vaccines. In northern Nigeria, where rotavirus coverage is estimated to be the lowest, poor information availability and access to immunization services were found to be main contributors to geographic clustering of low vaccination coverage [45]. Other barriers such as travel distance to health facilities, informal payments, and reliance on public facilities have been found to contribute to lower coverage for children in poorer households [46]. Evidence-based strategies such as communication and messaging campaigns [47,48], intensifying routine immunization activities (e.g. trainings, supportive supervision, outreach sessions, supply assurance, and monitoring), and using geographic information systems to identify low-coverage and underserved areas are all promising approaches for improving the effectiveness of future interventions [49] and reducing inequalities in potential outcomes.

Our rotavirus vaccination model for Nigeria has some important limitations. First, we assumed that vaccine efficacy does not vary across subpopulations. However, there is evidence of variability in efficacy based on income [41,50], geography [51], and nutritional status [52]. In addition, we used the same values for vaccine efficacy for the different vaccines despite slightly different results in the respective clinical studies [53,54]. The rationale for this decision is that no head-to-head clinical evaluation has occurred in Africa, making any differential vaccine performance assumption challenging, especially across subpopulations. Furthermore, we assumed that all vaccines had similar costs associated with delivery and administration. While the vaccines differ, we do not have sufficient evidence to distinguish delivery and administration costs by product. However, the purpose of this study was not to undertake a head-to-head economic comparison of these vaccine products. Rather, we demonstrate as our base case that the economic differences between a general vaccine and existing vaccines considered in this research are small, so policy makers should consider non-economic characteristics of each vaccine, such as cold chain and storage requirements and training health personnel. Finally, our scenarios showing the potential impacts of improving equity in vaccination coverage to not consider the associated costs of improving health infrastructure and increasing personnel to expand access to EPI vaccinations.

## Supporting information

S1 Appendix Excel file with aggregated input data, estimated from the 2013 Nigeria Demographic and Health Surveys (NDHS), used to build the impact cost-effectiveness model. The data from the 2013 NDHS underlying aggregates used in building the model are freely available at https://dhsprogram.com.
(XLSX)

S1 Fig Estimated impact and cost-effectiveness of ROTAVAC vaccination by region and wealth quintile. Mortality burden (deaths, a) and benefit (deaths averted, b) are expressed as rates per 1000 births over the first five years of life. Benefit is also expressed as mortality reduction (c) which is the percentage of burden prevented by vaccination. Incremental cost-effectiveness ratios (ICERs) are displayed in green (d).
(TIFF)

S2 Fig Estimated impact and cost-effectiveness of ROTASIIL vaccination by region and wealth quintile. Mortality burden (deaths, a) and benefit (deaths averted, b), expressed, are shown as rates per 1000 births over the first five years of life. Benefit is also expressed as mortality reduction (c) which is the percentage of burden prevented by vaccination. Incremental cost-effectiveness ratios (ICERs) are displayed in green (d).
(TIFF)

S3 Fig Estimated impact and cost-effectiveness of ROTARIX vaccination by region and wealth quintile. Mortality burden (deaths, a) and benefit (deaths averted, b), expressed, are shown as rates per 1000 births over the first five years of life. Benefit is also expressed as mortality reduction (c) which is the percentage of burden prevented by vaccination. Incremental cost-effectiveness ratios (ICERs) are displayed in green (d).
(TIFF)

S4 Fig Estimated impact and cost-effectiveness of ROTAVAC 5D vaccination by region and wealth quintile. Mortality burden (deaths, a) and benefit (deaths averted, b), expressed, are shown as rates per 1000 births over the first five years of life. Benefit is also expressed as mortality reduction (c) which is the percentage of burden prevented by vaccination. Incremental cost-effectiveness ratios (ICERs) are displayed in green (d).
(TIFF)

S5 Fig Estimated impact and cost-effectiveness of ROTASIIL LQ vaccination by region and wealth quintile. Mortality burden (deaths, a) and benefit (deaths averted, b), expressed, are shown as rates per 1000 births over the first five years of life. Benefit is also expressed as mortality reduction (c) which is the percentage of burden prevented by vaccination. Incremental cost-effectiveness ratios (ICERs) are displayed in green (d).
(TIFF)

S6 Fig Estimated incremental cost-effectiveness ratios for rotavirus vaccination for five rotavirus vaccines by rotavirus mortality burden for each region (a-e) and wealth quintile (f-g) subpopulation. (TIFF)

S1 Table Regional quintile estimates of rotavirus burden, rotavirus vaccine effectiveness, averted costs of illness, vaccination costs and cost-effectiveness for a cohort of children in Nigeria over the first five years of life. Socioeconomic subpopulations of children in each region were grouped into quintiles using an household asset-based index. ‘Average Nigeria perspective’ incremental cost-effectiveness ratio (ICER) base case estimates assumed Gavi subsidies for the first 5 years of vaccine introduction.
(XLSX)
