# Supplementary figures and images for: Effects of geographic and economic heterogeneity on the burden of rotavirus diarrhea and the impact and cost-effectiveness of vaccination in Nigeria
Source: PLoS One. 2020 May 29;15(5):e0232941. doi: 10.1371/journal.pone.0232941 (PMC7259699; doi:10.1371/journal.pone.0232941)

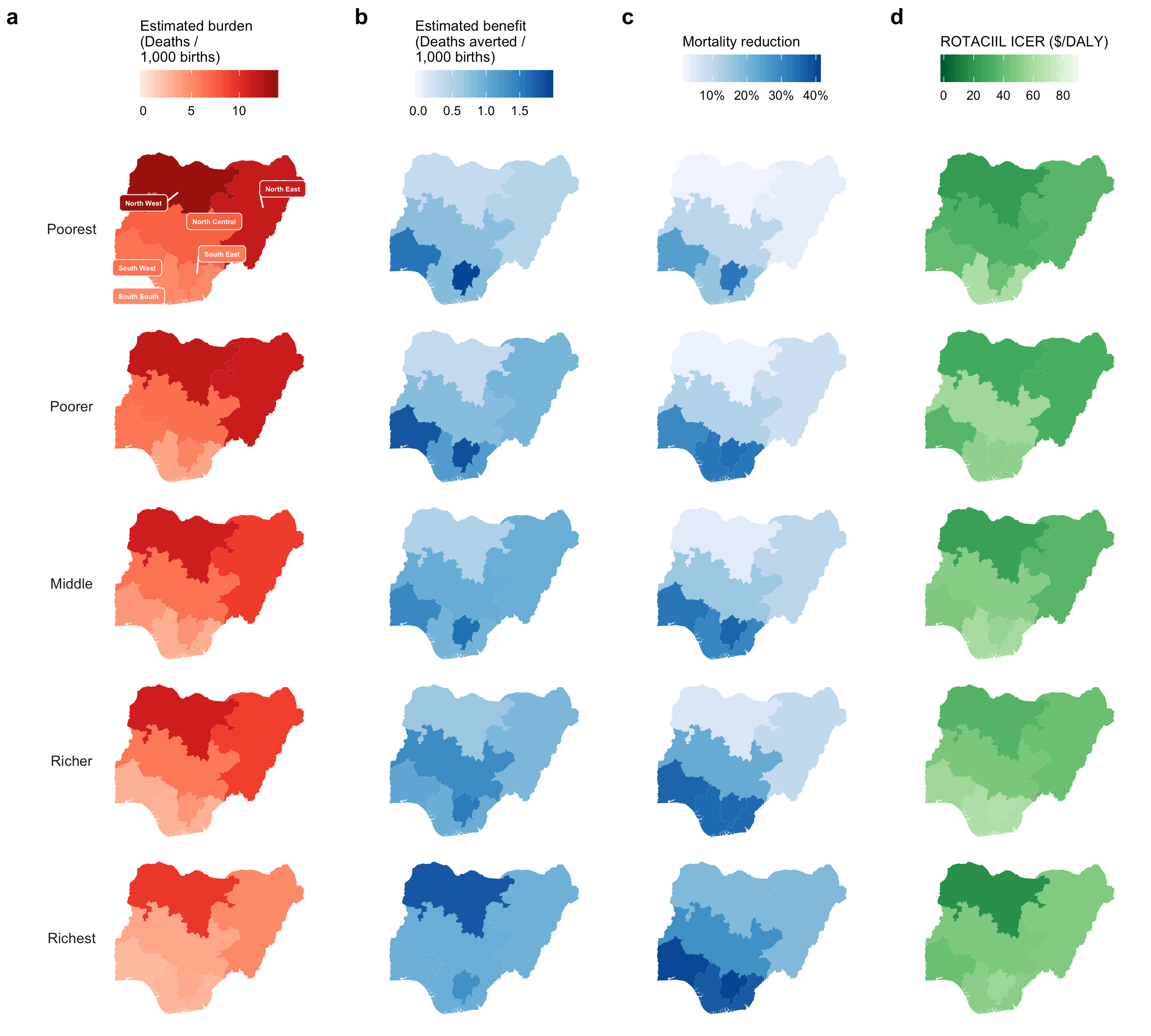

Supplement: S1 Fig — Mortality burden (deaths, a) and benefit (deaths averted, b) are expressed as rates per 1000 births over the first five years of life. Benefit is also expressed as mortality reduction (c) which is the percentage of burden prevented by vaccination. Incremental cost-effectiveness ratios (ICERs) are displayed in green (d). (TIFF) [file pone.0232941.s002.tiff]

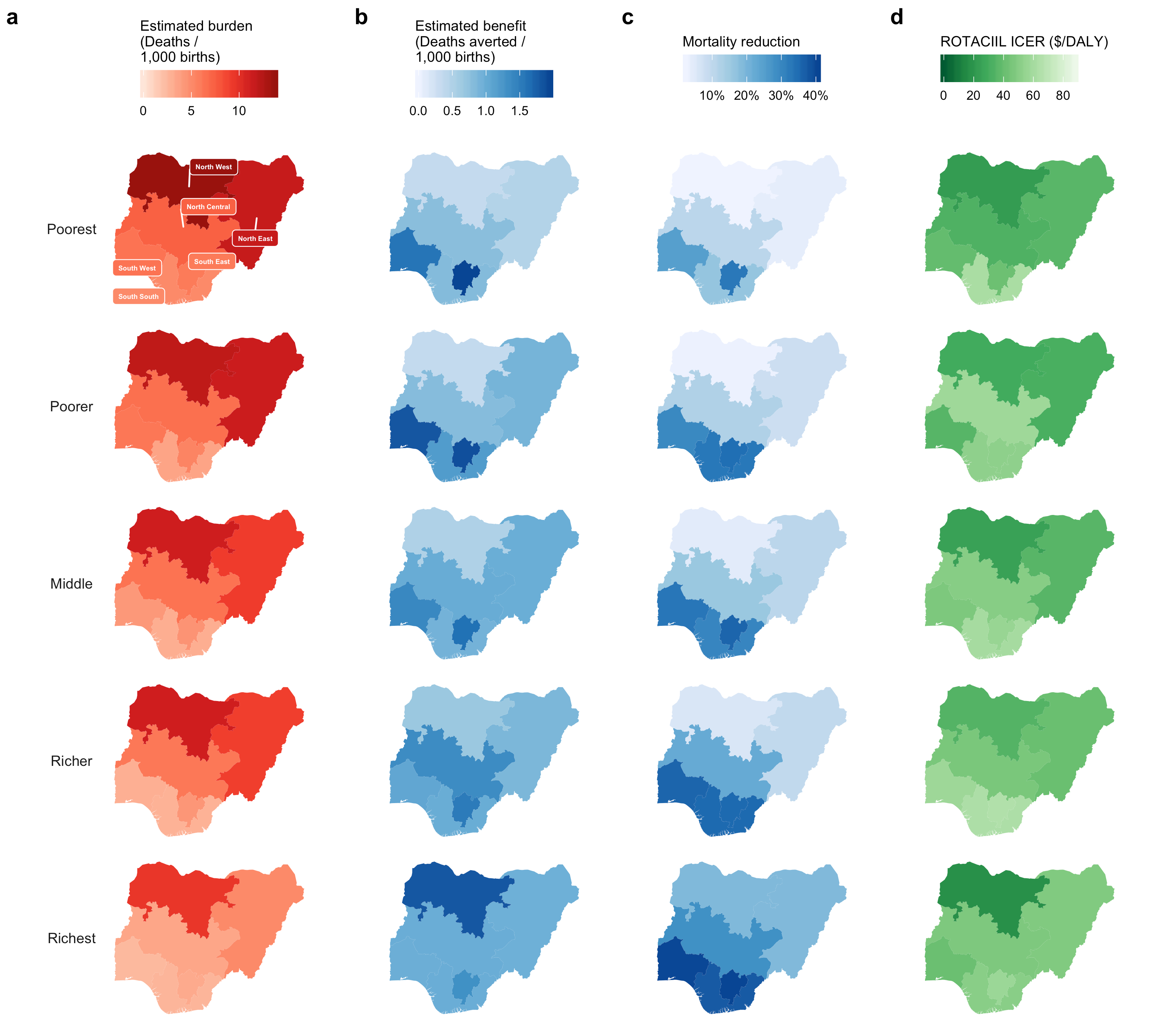

Supplement: S2 Fig — Mortality burden (deaths, a) and benefit (deaths averted, b), expressed, are shown as rates per 1000 births over the first five years of life. Benefit is also expressed as mortality reduction (c) which is the percentage of burden prevented by vaccination. Incremental cost-effectiveness ratios (ICERs) are displayed in green (d). (TIFF) [file pone.0232941.s003.tiff]

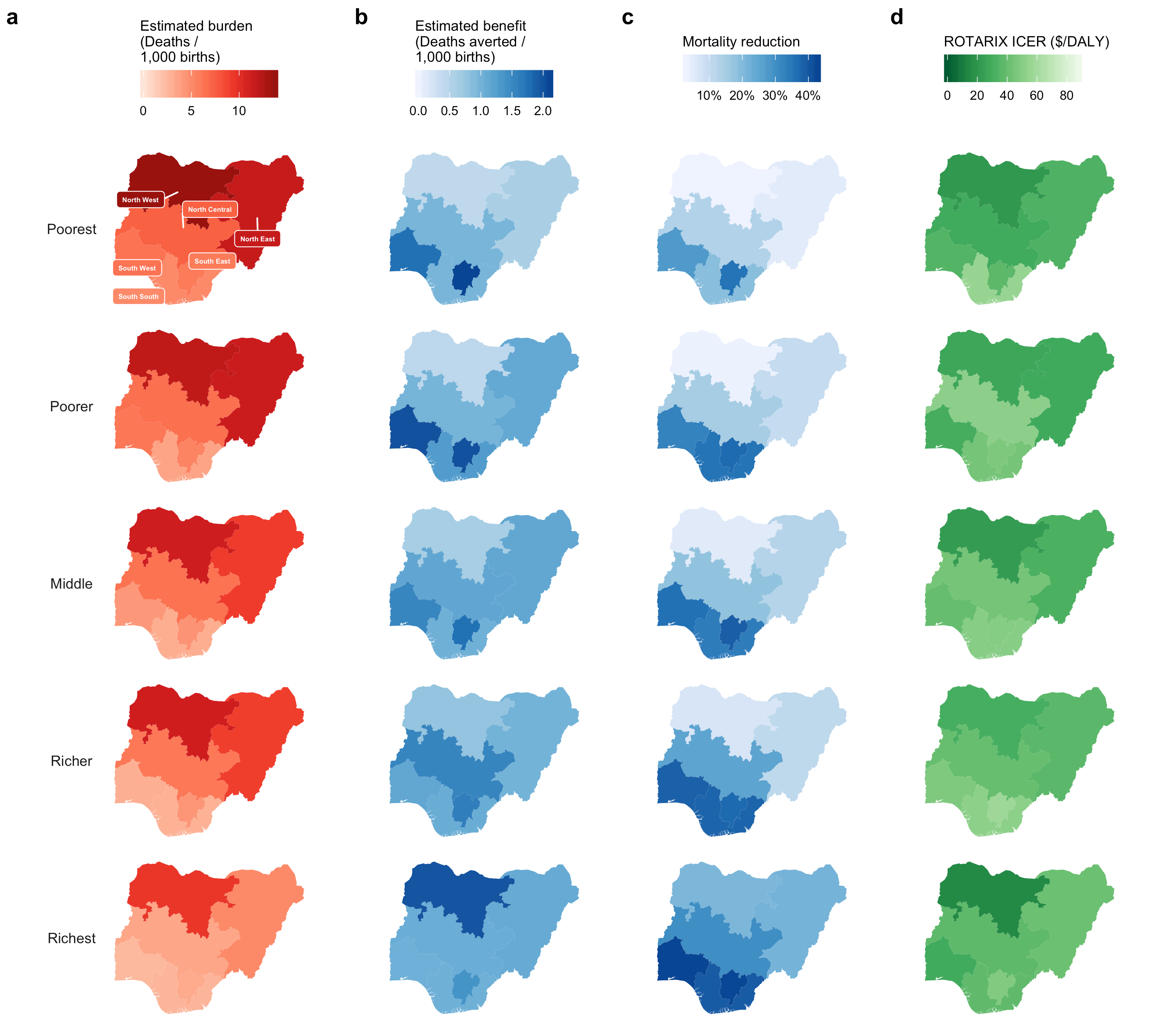

Supplement: S3 Fig — Mortality burden (deaths, a) and benefit (deaths averted, b), expressed, are shown as rates per 1000 births over the first five years of life. Benefit is also expressed as mortality reduction (c) which is the percentage of burden prevented by vaccination. Incremental cost-effectiveness ratios (ICERs) are displayed in green (d). (TIFF) [file pone.0232941.s004.tiff]

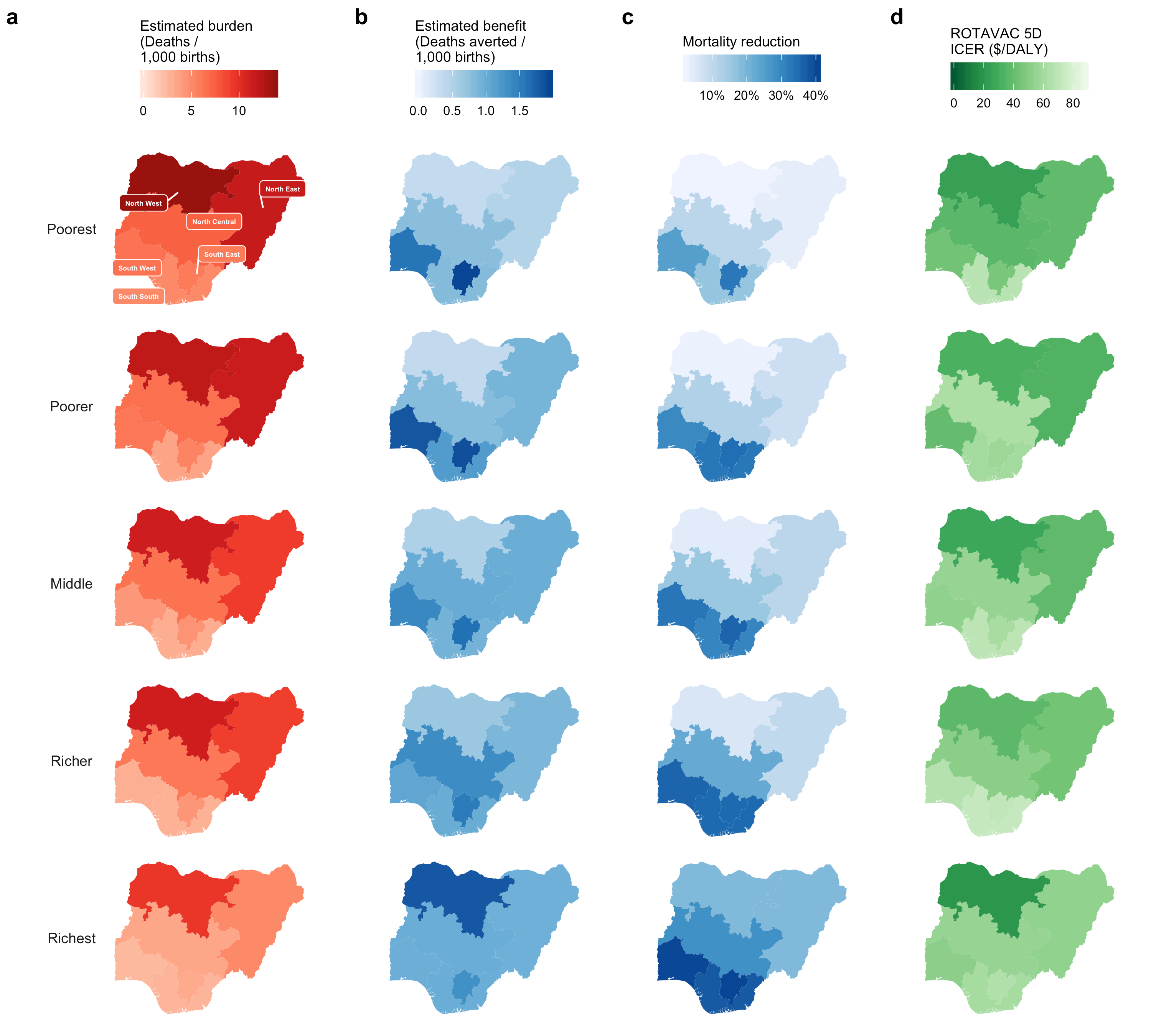

Supplement: S4 Fig — Mortality burden (deaths, a) and benefit (deaths averted, b), expressed, are shown as rates per 1000 births over the first five years of life. Benefit is also expressed as mortality reduction (c) which is the percentage of burden prevented by vaccination. Incremental cost-effectiveness ratios (ICERs) are displayed in green (d). (TIFF) [file pone.0232941.s005.tiff]

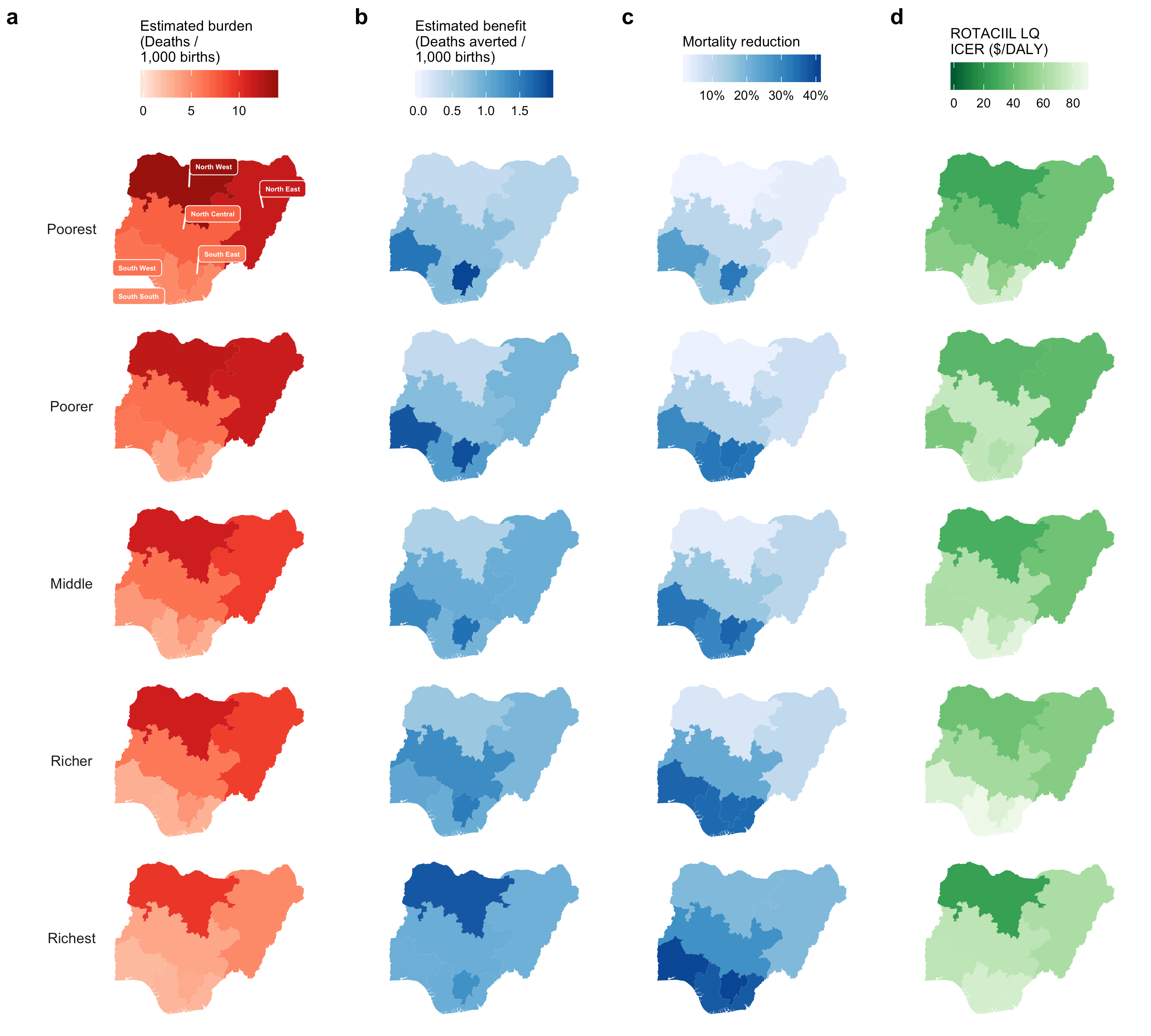

Supplement: S5 Fig — Mortality burden (deaths, a) and benefit (deaths averted, b), expressed, are shown as rates per 1000 births over the first five years of life. Benefit is also expressed as mortality reduction (c) which is the percentage of burden prevented by vaccination. Incremental cost-effectiveness ratios (ICERs) are displayed in green (d). (TIFF) [file pone.0232941.s006.tiff]

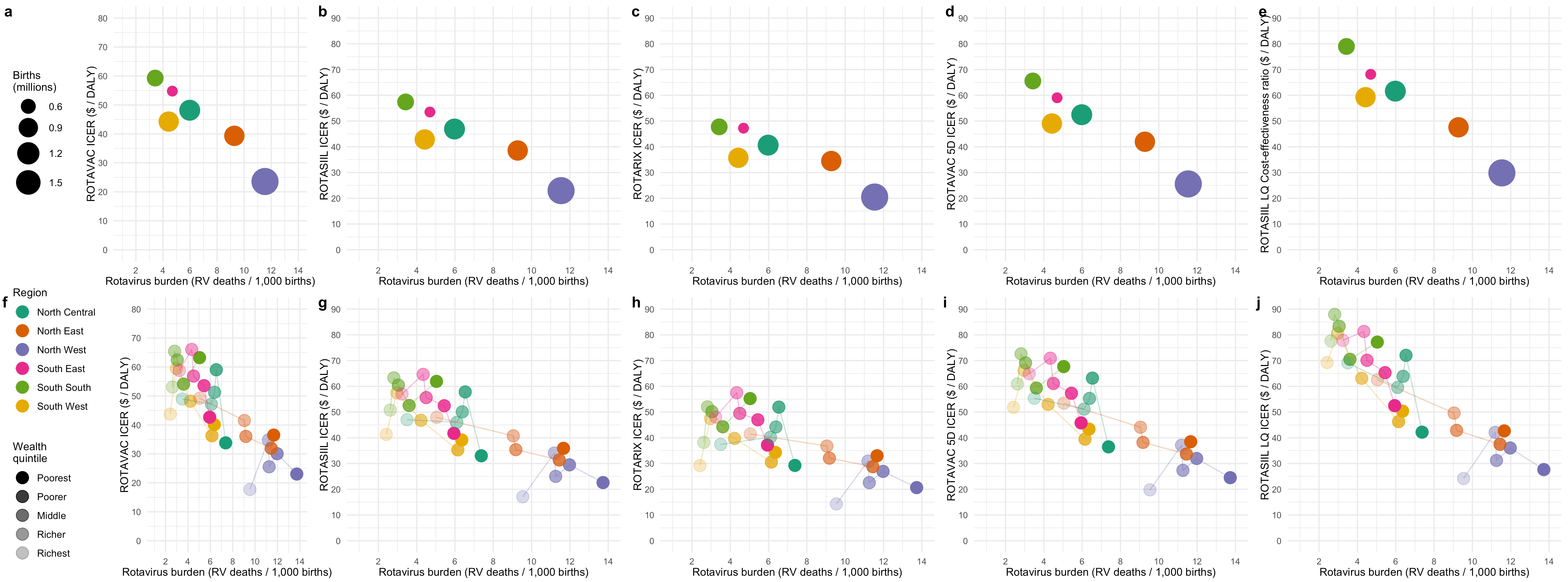

Supplement: S6 Fig — (TIFF) [file pone.0232941.s007.tiff]
